# A Rare Case Report of Lemierre Syndrome from the Anterior Jugular Vein

**DOI:** 10.5811/cpcem.2020.7.47442

**Published:** 2020-08-03

**Authors:** Nima Rejali, Marissa Heyer, Doug Finefrock

**Affiliations:** * Hackensack University Medical Center, Department of Emergency Medicine, Hackensack, New Jersey; † Hackensack Meridian School of Medicine, Department of Emergency Medicine, Hackensack, New Jersey

**Keywords:** Sepsis, septic emboli, thrombophlebitis, case report, Lemierre

## Abstract

**Introduction:**

Lemierre syndrome is a rare, potentially fatal, septic thrombophlebitis of the internal jugular vein. Treatment includes intravenous antibiotics for *Fusobacterium necrophorum*, the most common pathogen, as well as consideration for anticoagulation therapy.

**Case Report:**

A 27-year-old female presented with left-sided neck swelling and erythema. Computed tomography noted left anterior jugular vein thrombophlebitis and multiple cavitating foci, consistent with septic emboli. We report a rare case of Lemierre syndrome in which the thrombus was found in the anterior jugular vein, as opposed to the much larger internal jugular vein more traditionally associated with creating septic emboli.

**Conclusion:**

Based on an individual’s clinical symptoms, history, and radiologic findings, it is important for physicians to consider Lemierre syndrome in the differential diagnosis, as the condition may rapidly progress to septic shock and death if not treated promptly. The use of anticoagulation therapy remains controversial, and there is a lack of established standard care because the syndrome is so rare.

## INTRODUCTION

Lemierre syndrome is a rare septic thrombophlebitis of the internal jugular vein that will almost certainly result in mortality if proper treatment is not established quickly.^1^ It is commonly preceded by an oropharyngeal infection, such as pharyngitis, and is complicated by bacteremia with primarily anaerobic organisms of the oral flora.^1^ The most common pathogen associated with Lemierre syndrome is *Fusobacterium necrophorum*, a Gram-negative, anaerobic bacillus that inhabits the oropharynx.^2^ While the mechanism of how this generally noninvasive organism infiltrates mucosal surfaces is unknown, some research has pointed to viral or bacterial infections altering the pharyngeal mucosa, allowing the bacteria to invade parapharyngeal and carotid spaces via direct or venolympathic route.^3^ Affected individuals are often young (age 16–30 years) and initially present with sore throat, followed by neck pain and a neck mass.^4^ Patients require hospital admission for intravenous (IV) antibiotic administration to eradicate the infection.

## CASE REPORT

A 27-year-old female presented to the emergency department (ED) for swelling and redness of the left neck. She noted having a “pimple on her chin” two weeks prior, which she popped. A few days later, she developed swelling to her left neck and jaw. The swelling progressed and required emergent intubation and an intensive care unit admission at a nearby hospital for one week. Her treatment included IV antibiotics and steroids without surgical drainage. After extubation, she exhibited concern for proper treatment and left against medical advice (AMA). The next day she presented to our ED. The patient stated that she was also treated for a “lung infection,” but was unclear of the diagnosis. She noted that over the prior few days her “neck infection,” which initially improved, had increased swelling and redness. She denied any difficulty swallowing or speaking, fever, nausea, or vomiting. She was unsure of her diagnosis or specific antibiotic treatment and could not recall whether any cultures were obtained.

Upon arrival to the ED, the patient’s blood pressure was 107/68 milligrams of mercury with a pulse of 99 beats per minute and temperature of 98.2° Fahrenheit (36.8°Celsius). Her respiratory rate was 20 breaths per minute, and oxygen saturation on room air was 98%. Her height was 1.575 meters (5′2″) and weight was 83 kilogram (kg) (183 pounds) with a body mass index of 33.47 kg per meters squared (m^2^) (reference range 18.5–24.9 kg/m^2^).

On physical exam, the patient was oriented to person, place, and time; however, she appeared visibly dyspneic ambulating from the waiting area to exam room. She had a pressure ulcer on the lower lip (likely from endotracheal tube), and fluctuance, erythema, and tenderness to teeth numbered 18 and 19. There was a large area of erythema, induration, and warmth on the left mandible, approximately 10 × 6 centimeters (cm), with no central fluctuance. Exam was negative for elevation of the tongue, uvular deviation, pharyngeal edema or erythema, and brawny edema of the anterior neck. Patient’s pupils were equal, round, and reactive to light with extraocular motions intact.

Pulmonary/chest exam was notable for mild tachypnea with cough, as well as bilateral rales (right > left). She had no stridor, drooling, voice changes, or other concerning symptoms requiring emergent airway stabilization. The patient had normal rate and regular rhythm on cardiac exam, with no murmurs heard. She had a soft, non-tender abdomen with normal bowel sounds. There were no abnormal findings on neurological exam, and the patient’s skin was warm, dry, and non-diaphoretic.

Overall, suspicion for a serious medical illness upon initial presentation was high: the patient had recently been intubated for respiratory distress and follow-up management was challenged by the lack of a proper transition of care. Medical records were not available for review during her ED presentation, and she had left another hospital AMA the day prior. In addition to the patient’s obvious discomfort and fatigue, a detailed head, eyes, ears, nose, and throat exam revealed many abnormalities, most notably an intraoral abscess and significant redness and swelling to the neck concerning for large abscess vs cellulitis.

**CPC-EM Capsule**

What do we already know about this clinical entity?

Lemierre syndrome is a rare clinical diagnosis that carries high morbidity and mortality if not identified early.

What makes this presentation of disease reportable?

The disease usually presents with thrombus in the internal jugular vein. In our case, disease pathology was observed via the much smaller anterior jugular vein.

What is the major learning point?

If a patient presents with an active or recent neck infection and shortness of breath, Lemierre syndrome should be on the differential diagnosis.

How might this improve emergency medicine practice?

Early recognition and antibiotics, as well as early consultation with appropriate consultants, can improve patient outcomes with Lemierre syndrome.

The diagnostic evaluation included laboratory testing with blood cultures (Table 1). The patient was started on broad spectrum IV antibiotics: ampicillin/sulbactam and vancomycin, as methicillin-resistant *Staphylococcus aureus* was also considered given her recent hospital admission. A dental consultation was obtained and an incision and drainage of an abscess at tooth 19 was performed prior to computed tomography (CT) imaging. A CT soft tissue neck with IV contrast was ordered. A CT chest was also ordered to further evaluate her recent history of “lung infection,” fatigue with ambulation, and rales on pulmonary exam. Care coordination occurred with the radiologist to consider both pneumonia and pulmonary septic emboli. The radiologist recommended a traditional pulmonary embolus protocol study.

**Image 1:**
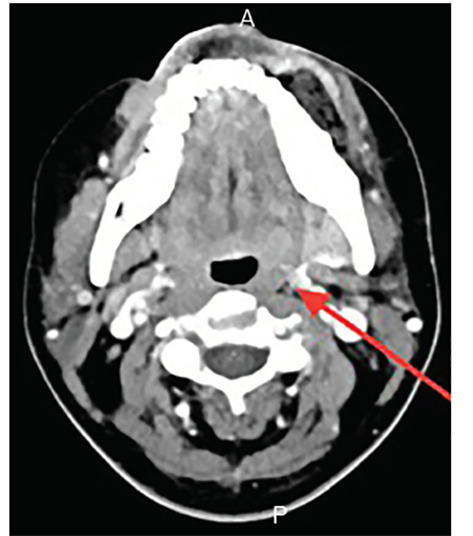
Computed tomography soft tissue neck with intravenous contrast demonstrating thickening in the left submandibular soft tissues concerning for cellulitis or the sequela of thrombophlebitis (arrow), with thrombus of the left anterior jugular vein and suspected thrombus of the superficial branches of the superficial facial vein branches and left perimandibular region.

**Image 2:**
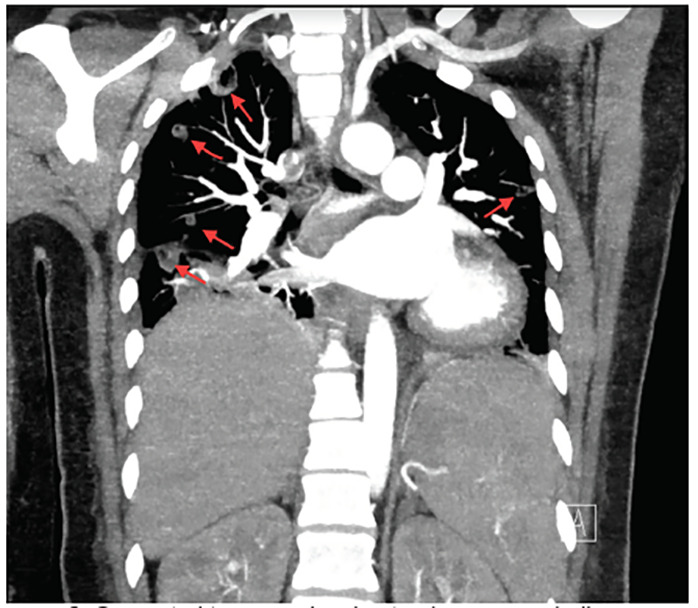
Computed tomography chest pulmonary embolism protocol, demonstrating numerous cavitating nodular consolidations (arrows), concerning for septic emboli.

CT findings were consistent with a diagnosis of Lemierre syndrome. CT neck images revealed a 3.2 cm area of ill-defined low density and gas in the left buccal perimandibular soft tissues, concerning for site of reported abscess status-post incision and drainage. There were numerous small areas of low attenuation overlying thickening in the left submandibular soft tissues, raising concern for possible cellulitis or thrombophlebitis sequelae (Image 1). Finally, thrombus of the left anterior jugular vein was visualized, as well as suspected thrombus of the superficial facial vein branches and left perimandibular regions. CT chest study revealed numerous cavitating nodular consolidations concerning for septic emboli given the patient’s history, in addition to a small right pleural effusion (Image 2). Pulmonology, vascular surgery, and infectious disease were consulted by the ED, and care was transitioned to the admitting hospitalist.

The patient was continued on IV antibiotics as an inpatient. Blood cultures remained negative throughout inpatient stay. Given a concern for endocarditis, a transthoracic echocardiogram was performed followed by a transesophageal echocardiogram. Both were negative for any signs of endocarditis or structural heart abnormalities. Vascular surgery did not recommend anticoagulation. The patient remained stable, transitioned to oral antibiotics, and was discharged home on a two-week course of sulfamethoxazole/trimethoprim and amoxicillin/clavulanic acid. During a follow-up appointment in primary care clinic one week after discharge, she remained afebrile without any new complaints. The plan was made to finish her oral antibiotic course and follow up with pulmonology in the clinic for a repeat CT chest study and re-evaluation.

## DISCUSSION

As documented in the literature, Lemierre syndrome is classically associated with thrombus of the internal jugular vein; however, there are only limited case reports demonstrating involvement of the smaller anterior jugular vein.^5^ Although otherwise healthy, these patients may appear acutely ill with tachycardia, tachypnea, hypotension, and oxygen saturation less than 95%.^6^ Typical lab findings of Lemierre syndrome are notable for neutrophilic leukocytosis and signs of organ insult to the affected organs.^6^ CT soft tissue neck with IV contrast best confirms septic thrombophlebitis, while CT chest may show multiple necrotic cavitary lesions, characteristic of Lemierre syndrome.^2^,^3^ Septic emboli have been reported in the lungs, kidneys, liver, joints, peritoneum, and brain.^3^

*F. necrophorum* is usually susceptible to beta-lactam antibiotics, such as penicillin, as well as protein and deoxyribonucleic acid synthesis inhibitors, such as clindamycin, and metronidazole, respectively, but is resistant to macrolides.^7^ Additionally, some *F. necrophorum* are capable of producing beta-lactamase, and develop resistance to beta-lactam antibiotics.^8^ As a result, patients are generally treated with metronidazole, carbapenem, or a penicillin/beta-lactamase inhibitor combination. Although the overall incidence is about 0.6–3.6 cases per million, incidence rates appear to be increasing likely because of antibiotic resistance.^7^ Decisions on pharmacological treatment are case-dependent.^2^,^9^

The use of anticoagulation therapy has been controversial and varies on an individual basis. Since Lemierre syndrome is rare, it is essentially impossible to collect direct outcome measures based on anticoagulation therapy.^10^ Anticoagulation therapy, in the case of Lemierre syndrome, is aimed at preventing possible life-threatening consequences of septic thromboembolism, such as respiratory failure, septic arthritis, and retrograde thrombophlebitis extending to intracranial sinuses.^10^ Because the disease is an inflammatory process, there is a possibility that resolution of inflammation could cause spontaneous improvement of the thrombosis.^11^ Recanalization of the internal jugular vein has been observed in some patients; however, other authors have reported that recanalization is generally uncommon.^11^,^12^

Successful management depends on an initial high index of suspicion and a multidisciplinary-team treatment approach. 4

## CONCLUSION

Lemierre syndrome is typically characterized as a septic thrombophlebitis of the internal jugular vein, or less commonly the anterior jugular vein. The condition is frequently attributed to infection with *F. necrophorum* and may progress to septic shock if left untreated. Since the syndrome is so rare, there is no established standard of care with regard to antibiotic treatment. Implementation of anticoagulation therapy continues to remain a controversial topic that varies on an individual basis. Due to the high risk of mortality associated with the disease, it is important for physicians to consider Lemierre syndrome as part of a differential diagnosis based on the presentation of the patient’s clinical symptoms, history, and radiologic findings.

## Figures and Tables

**Table:** Complete blood count and complete metabolic panel of patient with Lemierre syndrome.

	Result	Reference range
White blood cells	18.0×10^3^/mcL	4.5–13.5×10^3^/mcL
Hemoglobin	11.9 gm/dL	12.0–15.0 gm/dL
Hematocrit	36.7%	34.0–43.0%
Platelets	396×10^3^/mcL	135–430×10^3^/mcL
Neutrophil %	94.1%	40.0–75.0%
Sodium (Na^+^)	140 mmol/L	138–145 mmol/L
Potassium (K^+^)	4.5 mmol/L	3.4–4.7 mmol/L
Chloride (Cl^−^)	103 mmol/L	96–109 mmol/L
Carbon dioxide (CO_2_)	26 mmol/L	20–28 mmol/L
Blood urea nitrogen	10 mg/dL	7.0–16.8 mg/dL
Creatinine	0.5 mg/dL	0.5–1.1 mg/dL
Glucose	166 mg/dL	60–100 mg/dL
Lactic acid	1.9 mmol/L	< 2.0 mmol/L

*mcL*, microliters; *gm*, gram; *dL*, deciliter; *mmol*, millimole; *L*, liter; *mg*, milligram.
